# Integrative genomic and transcriptomic analysis reveals immune subtypes and prognostic markers in ovarian clear cell carcinoma

**DOI:** 10.1038/s41416-022-01705-w

**Published:** 2022-01-18

**Authors:** Shuang Ye, Qin Li, Yutuan Wu, Wei Jiang, Shuling Zhou, Xiaoyan Zhou, Wentao Yang, Xiaoyu Tu, Boer Shan, Shenglin Huang, Huijuan Yang

**Affiliations:** 1 Department of Gynecologic Oncology, Fudan University Shanghai Cancer Center, Shanghai, China; 2 Department of Oncology, Shanghai Medical College, Fudan University, Shanghai, China; 3 Fudan University Shanghai Cancer Center, and the Shanghai Key Laboratory of Medical Epigenetics, Institutes of Biomedical Sciences, Fudan University, Shanghai, China; 4 Department of Pathology, Fudan University Shanghai Cancer Center, Shanghai, China

**Keywords:** Ovarian cancer, Prognostic markers

## Abstract

**Background:**

We performed an integrative genomic and transcriptomic profiling to identify molecular subtypes and prognostic markers with special focus on immune-related pathways.

**Methods:**

Totally, 50 Chinese patients were subjected to targeted next-generation sequencing and transcriptomic sequencing.

**Results:**

Two distinct subgroups were identified as immune (22.0%) and non-immune (78.0%) based on the immune-pathway related hierarchical clustering. Surprisingly, patients with immune subtype had a significantly worse survival. The prognostic capacity was validated in external cohorts. The immune group had higher expression of genes involved in pro-inflammation and checkpoints. PD-1 signalling pathway was enriched in the immune subtype. Besides, the immune cluster presented enriched expression of genes involved in epithelial-mesenchymal transition, angiogenesis and PI3K-AKT-mTOR signalling, while the non-immune subtype had higher expression of metabolic pathways. The immune subtype had a higher mutation rate of PIK3CA though significance was not achieved. Lastly, we established a prognostic immune signature for overall survival. Interestingly, the immune signature could also be applied to renal clear cell carcinoma, but not to other histologic subtype of ovarian cancer.

**Conclusions:**

An immune subtype of OCCC was identified with poor survival and enrichment of PD-1 and PI3K-AKT-mTOR signalling. We constructed and validated a robust prognostic immune signature of OCCC patients.

## Background

Ovarian clear cell carcinoma (OCCC), as s histologic subtype of epithelial ovarian cancer, has distinctive clinical and biological behaviour. It has a variable geographical distribution with the highest prevalence in Asian women [1]. In the past seven years, we tried to describe the biological behaviour and clinicopathological characteristics of Chinese OCCC patients [2–9]. OCCC represents a great challenge and unmet need given its disease aggressiveness and chemotherapy resistance [10, 11]. Novel biological agents are urgently needed. Endeavours have been made to clarify the molecular features of OCCC to explore therapeutic targets from both genomic and transcriptomic levels [12–16]. Genomic landscape of OCCC has been characterised in a number of sequencing studies with modest sample sizes [13–15]. Driver mutations in ARID1A, PIK3CA, and deregulated PI3K/AKT/mTOR and RAS/RAF/REK pathways are more commonly reported in OCCC [13–15]. Gene expression studies reveal up-regulation of hepatocyte nuclear factor 1-beta and oxidative stress-related genes [17, 18]. A recent study has identified two transcriptomic subtypes named as epithelial- and mesenchymal-like with different prognosis [16].

In spite of these efforts, there are still no effective targeted treatments for OCCC currently. Genomic studies have presented mutational similarities between ovarian and renal clear cell carcinoma, where the anti-angiogenic agent sunitinib is approved in clinical setting [19]. However, in recently published GOG 254 trial, sunitinib, a multi-kinase inhibitor targeting vascular endothelial growth factor receptor (VEGF-R), showed minimal activity in the second- and third-line treatment in OCCC as a single agent [20]. In another phase II trial of pembrolizumab (anti-PD-1 antibody) for recurrent ovarian cancer (KEYNOTE-100), the response rate of clear cell carcinoma (*N* = 19) was 15.8% compared to 8% for the entire cohort (*N* = 300) [21]. Immunotherapy might be promising in OCCC and further results are awaited including durvalumab (NCT03405454), a combination treatment of nivolumab and ipilimumab (NCT03355976).

In the present study, we conducted genomic and transcriptomic analyses in a well-annotated cohort of Chinese OCCC patients to explore possible prognostic biomarkers and different subtypes, which might shed light on subtype-tailored treatment. Considering the promising role of immunotherapy, we specifically looked for a defined immune gene expression signature in transcriptomic analysis to develop immune classifiers and candidate biomarkers of immune treatment.

## Methods

### Study population and datasets

A total of 50 archived tumours were collected and sequenced from Fudan University Shanghai Cancer Center after obtaining the institutional review board approval (050432-4-1212B) from 2014 to 2018. In our institution, all the patients are asked whether they are willing to donate their tumour for research purpose and sign the informed consent. In the current study, the hematoxylin & eosin-stained slides were reviewed by an experienced gynecology-dedicated pathologist to confirm the diagnosis. Mixed histology was excluded. Clinicopathologic information and survival outcomes were abstracted from medical records. The following data was extracted: the age at diagnosis, date and type of primary surgery, International Federation of Gynecology and Obstetrics (FIGO) stage at initial diagnosis, residual disease, platinum-free interval (the time interval from completion of the last platinum-based chemotherapy to disease recurrence), time of disease progression or recurrence, and tumour status at last contact. Patients were considered as platinum-sensitive if the platinum-free interval was more than 6 months. Progression-free survival (PFS) and overall survival (OS) was defined as the time interval from the date of the primary surgery to the date of first recurrence and death or last contact, respectively.

We retrospectively analysed the gene expression profiles and clinical parameters of ovarian cancer patients from five public cohorts, including three microarray datasets and two RNA-Seq dataset from The Cancer Genome Atlas (TCGA). Only patients with relevant clinical information were included. The OCCC datasets from our institution was used as the training set, and we extracted genome-wide gene expression microarray data using OCCC tissues from GSE73614 [22] and GSE65986 [12] as validation datasets. Moreover, another four datasets from different platforms were used as independent validation sets including TCGA-KIRC, TCGA-OV (ovarian high-grade serous cancer), GSE63885 [23] (ovarian serous cancer) and GSE73614 (ovarian endometrioid cancer). In microarray analysis, probe IDs were mapped to gene symbols according to the corresponding annotation file, and expression measurements of all probes related to a same gene were averaged to obtain a single value.

### Genomic sequencing

The formalin-fixed paraffin-embedded (FFPE) tumour samples and peripheral blood were obtained from 50 patients. The capture-based targeted sequencing was performed at Burning Rock Biotech laboratory (Guangzhou, China), which is a College of American Pathologists (CAP)-accredited and Clinical Laboratory Improvement Amendments (CLIA)-certified clinical laboratory.

#### DNA extraction

The QIAamp Circulating Nucleic Acid Kit (Qiagen, Hilden, Germany) and the QIAamp DNA FFPE Tissue Kit (Qiagen, UK) were used to extract normal DNA from blood and tumour DNA from FFPE tumour samples, respectively, according to the manufacturer’s instructions. The Qubit 2.0 fluorometer and the Qubit dsDNA HS Assay Kit (Life Technologies, Carlsbad, USA) were used to measure DNA concentration.

#### Next-generation sequencing (NGS) library construction and sequencing

DNA shearing was performed using the M220 Focused-ultrasonicator (Covaris, USA), followed by end repair, phosphorylation and adaptor ligation. Fragments with size of 200–400 bp were selected by the Agencourt AMPure XP beads (Beckman Coulter, USA), followed by hybridisation with capture probes baits, hybrid selection with magnetic beads and PCR amplification. Target capture was performed with the OncoScreen Plus panel (Burning Rock Biotech, China) consisting of 520 cancer related genes (Supplementary Table S1), spanning 1.6 MB of human genome. The whole exons of 312 genes and critical exons, introns and promoter regions of the remaining 208 genes were captured. The commonly mutant genes in OCCC [24] were covered in the 520-gene panel. DNA quality and fragment size were assessed by Bioanalyzer 2100 (Agilent, USA). The indexed samples were sequenced on an Illumina NextSeq 500 paired-end system (Illumina, Inc., USA).

#### Sequence analysis

The paired-end reads were mapped to the human genome (hg19) by a Burrows-Wheeler aligner v.0.7.10 [25]. Local alignment optimisation, variant calling, and annotation were performed with the Genome Analysis Toolkit (GATK) v.3.2 [26] and VarScan v.2.4.3 [27]. DNA translocation analysis was performed with Factera v.1.4.3 [28]. The variants were filtered with the VarScan filter pipeline, and loci with depths of less than 100 were filtered out. Germline mutations were also filtered out by sequencing matched white blood cells from the samples. Base-calling in plasma and tissue samples required at least eight supporting reads for single nucleotide variations (SNV) and five supporting reads for insertion-deletion variations (INDEL), respectively. Variants with population frequencies of over 0.1% on the Exome Aggregation Consortium (ExAC), 1000 Genomes, dbSNP, and ESP6500SI-V2 databases were grouped as single-nucleotide polymorphisms (SNPs) and excluded from further analysis. The remaining variants were annotated with ANNOVAR [29] (2016–02–01 release) and SnpEff v.3.6 [30]. The tumour mutation burden (TMB) was defined as the number of somatic mutations excluding copy number variations (CNV), fusions and large genome rearrangement (LGR) per mega base of genome examined. To be more specific, the mutations counted include missense, synonymous, frameshift, splice site and indel mutations whereas the genomic regions examined include all coding sequences extending 20 bp into the introns. The kinase domains of EGFR and ALK genes were excluded for TMB calculation. Thus, the total examined regions were 1.26 M for 520-gene panel. The 520-gene panel based TMB was proved be positively correlated with whole-exome sequencing derived TMB [31].

### RNA sequencing

#### RNA isolation and RNA-seq

Total FFPE RNAs were isolated by RNAstorm FFPE RNA Isolation Kit (Cell Data Sciences, USA). Total RNAs were treated with DNase I (NEB) to remove DNA before constructing the RNA-seq libraries. Strand-specific RNA-seq libraries were prepared using the SMART cDNA synthesis technology (Clontech, USA). The cDNA was pre-amplified and the ribosomal and mitochondrial cDNA were depleted by CRISPR/Cas9 technology. Purified dsDNA was further subjected to 13 cycles of PCR amplification. The libraries were quality controlled with Qubit (Thermo Fisher Scientific, USA) and Qsep100 (BiOptic, China) and sequenced by the Illumina sequencing platform (Nova) on a 150 bp paired-end run.

#### Processing RNA-seq data

FastQC (Babraham Bioinformatics Institute) was used to check the sequencing quality, and high-quality reads were mapped to human reference genome (hg38) along with the gene annotation data (genecode v29) from the Genecode database using STAR (v2.5.3a) [32]. Raw read counts per gene were obtained using featureCount [33]. Transcripts per million (TPM) values were calculated with normalisation on a total number of counted reads.

#### Identification of immune clusters and integrative analysis of molecular features

We downloaded 17 immune-related pathways from the ImmPort database (https://immport.niaid.nih.gov). Protein-coding gene expression profiles were transferred to immune-related pathways expression levels using the single sample gene set enrichment analysis GSEA (ssGSEA) tool, and ssGSEA scores were z-score normalised. Clustering was performed using R heatmap package for ssGSEA z-score matrix (clustering method = average). The xCell algorithm [34] was used to calculate 64 cell types score using transcriptomic data. Differential expression analysis was performed between immune and non-immune group, and then GSEA was executed against hallmarks and reactome signature gene set from MSigDB database v6.2 database with dataset ranked from LogFC (http://software.broadinstitute.org/gsea/msigdb). The immune-related genes for display were obtained from a previous study [35].

#### Construction and validation of the prognostic model of OCCC

To build the prognostic model of OCCC patients based on the protein-coding gene expression profiles, differential expression analysis between immune group and non-immune group was performed. Genes with *P* value < 0.05 and logFC > 1.5 were identified as differentially expressed genes (DEGs). Then we used a Lasso-regularised Cox proportional hazard model with the glmnet package (version 2.0–5) [36] to select and sort the statistically significant clinical features. We performed a tenfold cross-validation on the training set to calculate the weight of LASSO penalty (denoted as lambda). The lambda = −2 was used for feature selection. The following formula based on a combination of Lasso-cox coefficient and gene expression was used to calculate the risk score: Model: Risk Score=BiSi where k, βi, Si represent the number of signature genes, the coefficient index, and scaled gene expression level, respectively. Univariate Cox analysis and forest visualisation were performed by R package survival and ezcox (https://github.com/ShixiangWang/ezcox). For survival analyses, patients were dichotomised into two groups: low versus high-risk score by median value. Next, the Kaplan–Meier curve and log-rank test was used for survival analysis.

#### Other analysis

Principal component analysis was performed by the R package factoextra. Time-to-event distributions were estimated using the Kaplan–Meier (KM) method and compared using the log-rank test. Volcano plots were plotted using R package ggpubr. The Wilcox test was used to compare various experimental groups. *P* values < 0.05 were considered statistically significant.

## Results

### Patient characteristics

A total of 50 patients from our institution were included as the training set (Table 1). Age of the patients ranged from 26 to 79 years and the median age was 52 years. Of all, 51.0% (25/49) were at stage I, 20.4% (10/49) were at stage II, 24.5% (12/49) were at stage III, and 4.1% (2/49) were at stage IV. Concerning platinum response, 22.4% (11/49) patients were platinum-resistant and 77.6% (38/49) were platinum-sensitive, respectively. During the study period, recurrence and death was observed in 53.0% (26/49) and 36.7% (18/49) of the patients, respectively.

**Table 1:** Patient characteristics.

	N(%)	Immune (*n* = 11)	Non-immune (*n* = 39)	*P* value^a^
Age (median 51.5, mean 50.6)				
Age > =52	25 (50.0%)	3	22	0.088
Age <52	25 (50.0%)	8	17	
Personal history of cancer				
Yes	10 (20.0%)	3	7	0.798
No	40 (80.0%)	8	32	
Family history of cancer				
Yes	11 (22.0%)	3	8	0.947
No	39 (78.0%)	8	31	
FIGO stage				
Stage I	25 (51.0%)	3	22	0.136
Stage II + III + IV	24 (49.0%)	7	17	
Residual disease				
No residual disease	41 (83.7%)	8	33	0.725
With residual disease	8 (16.3%)	2	6	
Platinum-response				
Platinum sensitive	38 (77.6%)	6	32	0.096
Platinum resistant	11 (22.4%)	5	6	

^a^Chi-square test.

We further extracted sample annotated as OCCC from public database: GSE73614 (Caucasian, *n* = 37) [22] and GSE65986 (Japanese, *n* = 25) [12]. In the GSE73614 cohort, the median age of the patients was 64 years old with a range from 41 to 88. Regarding tumour stage, 35.0% were at stage I, 19% were at stage II, 41% were at stage III, and 2% were at stage IV. For survival analysis, only OS information was available for the cohort. In the GSE65986 cohort, the majority of the patients (80%) presented with early stage (I+II) disease. Therefore, only five (20%) patients had disease recurrence, which leads to immature survival data.

### Two subtypes identified by transcriptomic analysis of OCCC

**Fig. 1:**
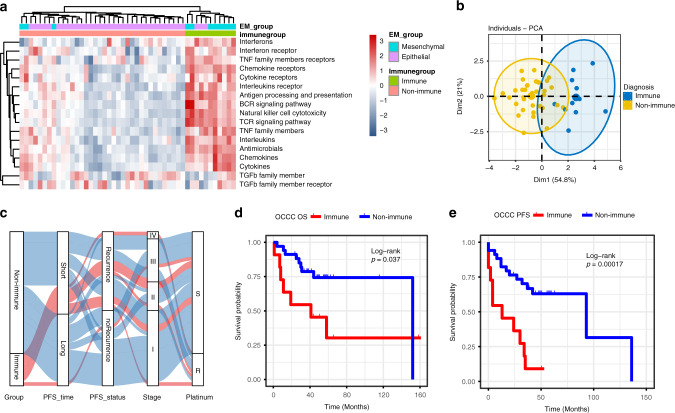
Immune-pathway-based subtyping of ovarian clear cell carcinoma (OCCC) and clinical implications. **a** Immune-pathway-based clustering results. Heatmap of normalised enrichment ssgsea scores using hierarchical clustering shows two distinct immune-pathway expression patterns, and the samples are labelled with the k-means cluster category shown at the top of the heatmap (immune, non-immune). **b** Two-dimensional scatterplot of principal component analysis. ssgsea scores were used from the immune and non-immune group. Clusters are indicated in light yellow and light blue. The percentage of the overall variation accounted for by principal components 1 (*x*-axis) and 2 (*y*-axis) is indicated for each axis. **c** Alluvial diagram showing the distribution of OCCC patients by cluster, progression-free survival (PFS) and status, tumour stage and response to platinum-based chemotherapy. Cluster Groups are: the immune group (red) and the non-immune (blue). PFS time is classified as: long (PFS_time > median) and short (PFS_time < median). For platinum response, R means resistant and S means sensitive. **d** Kaplan–Meier curves for overall survival (OS). **e** Kaplan–Meier curves for PFS. PFS = progression-free survival, OS = overall survival.

We performed immune-pathway related hierarchical clustering and identified two distinct subgroups (Fig. 1a). One subgroup had a significantly higher expression of the 15 immune-related pathways than the other (Fig. 1a, b). Thus, the two subgroups were designated as the immune (22.0%, 11/50) and non-immune (78.0%, 39/50), respectively. We compared the two subtypes in terms of clinical features (Table 1). The median age of the patients with immune and non-immune subtype was 50 and 52 years old, respectively. However, no significance was achieved. Similarly, patients with immune subtype tended to be platinum-resistant compared to those with non-immune subtype (45.5% vs. 15.8%), albeit with no significance. The two groups were quite comparable concerning tumour stage and residual disease after debulking surgery. From the Alluvial diagram (Fig. 1c), we can clearly see that the patients in the immune group had a higher recurrence rate and shorter survival than those in the non-immune group. Again, the Kaplan–Meier curves (Fig. 1d, e) illustrated that the immune subtype was related to worse survival including both PFS (*P* < 0.001) and OS (*P* = 0.037).

We further evaluated the prognostic capacity of the subtype classification in the two independent external cohorts (GSE73614, GSE65986) (Supplementary Fig. S1). Patients with immune subtype tended to have worse OS in both populations, though significance was not achieved (Supplementary Fig. S1A, B). Given the small sample sizes and outcome events, we amalgamated the two cohorts to increase the statistical power and found that the two OS curves were significantly separated (Supplementary Fig. S1C). More interestingly, the prognostic capacity of immune/non-immune subtype was further validated in renal clear cell carcinoma in the TCGA data (TCGA: KIRC) (Supplementary Fig. S2A). On the contrary, the classification could not be reproduced in patients with ovarian endometrioid (Supplementary Fig. S2B) or high-grade serous carcinoma (Supplementary Fig. S2C).

We sought to apply the previous epithelial- vs. mesenchymal-like subtype classification to our dataset according to the previous report [16]. We found that eight of eleven patients with immune subtype were classified as mesenchymal-like (Fig. 1a). Univariate and multivariate Cox regression analyses were performed to compare the prognostic impact of the two classifications (Supplementary Table S2). The results revealed that patients with epithelial subtype had significantly better PFS than those with mesenchymal counterpart. However, statistic significance was not achieved concerning OS, though tendency was noted.

### The two subsets displayed different immune profiles and signalling pathways

**Fig. 2:**
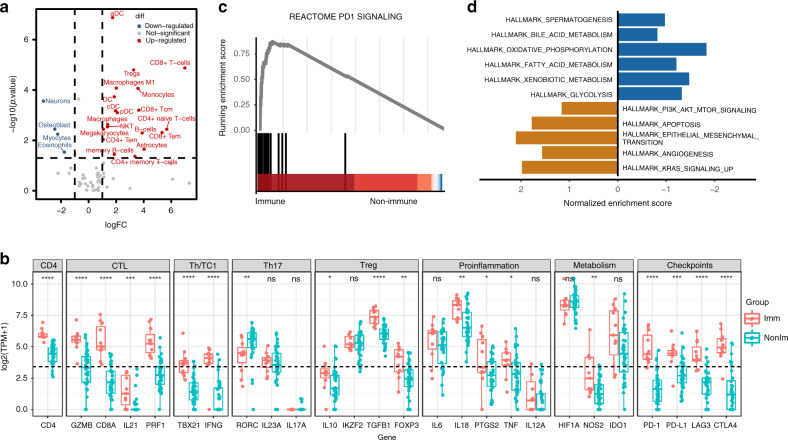
Comparison of features between the immune and non-immune group. **a** Volcano plot showing significantly differentially expressed xCell signatures. The *x*‐axis highlights the fold changes (log2 scale) (downregulated genes, negative values), whereas the *y*‐axis indicates the level of statistical significance (shown in −log10 format). Red and blue colours indicate up- and downregulated signatures, respectively. **b** Comparison of expression levels of immune-related genes including CD4, cytotoxic T lymphocyte (CTL), helper T cell 1/cytotoxic T cell 1 [TH1/TC1], TH17, regulatory T cells (Treg), proinflammation, metabolism, and checkpoint between immune and non-immune group. The graphs display values for each patients, and statistically significant differences between two groups using the Wilcox test. **c** A representative gene set enrichment analysis plot showing the programmed cell death protein 1 (PD-1) signalling pathway in the immune versus non-immune samples. **d** Pre-ranked Gene set enrichment analysis (GSEA) using ‘Hallmark’ gene sets showed enrichment of pathways. Normalised enrichment scores (NES) for signature gene sets representing RNA-seq data from protein coding genes of immune group or non-immune group. Abbreviations: ns= non-significant.

Using transcriptomic data, we next calculated the 64 cell types score by the xCell algorithm to explore the relationship between immune subgroup and immune cell infiltration. We found that the immune group had a significantly higher level of immune cells including CD8+ T cells, regulatory T cell (Treg), Macrophages M1, monocyte and dendritic cells (DC) (Fig. 2a). Aside from this, we noted that the immune-related genes involving CD4, cytotoxic T lymphocyte (CTL), helper T cell/ cytotoxic T cell 1 and Treg (TGF-β1 and FOXP3) were significantly upregulated in the immune cluster (Fig. 2b). Besides, the immune subtype showed enriched expression of genes involved in pro-inflammation (IL-18, PTGS2, TNF), metabolism (NOS2) and checkpoints (PD-1, PD-L1, LAG3, CTLA4) with statistic significance (Fig. 2b). As a representative, Fig. 2c shows the GSEA plot showing the PD-1 signalling pathway enrichment in the immune subtype compared to the non-immune counterpart. By pre-ranked GSEA using ‘Hallmark’ gene sets, we displayed that the two subtypes showed apparently different patterns of pathway enrichment (Fig. 2d). The immune cluster presented enriched expression of genes involved in apoptosis, epithelial-mesenchymal transition, angiogenesis, PI3K-AKT-mTOR and KRAS signalling. In contrast, the non-immune subtype had higher expression of metabolic pathways, including fatty acid, glucose, xenobiotic metabolism, and oxidative phosphorylation. Based on these, we postulated that the patients with immune subtype might be potential candidates for immunotherapy.

### Genomic landscape of OCCC and significantly mutated genes in the two immune subtypes

**Fig. 3:**
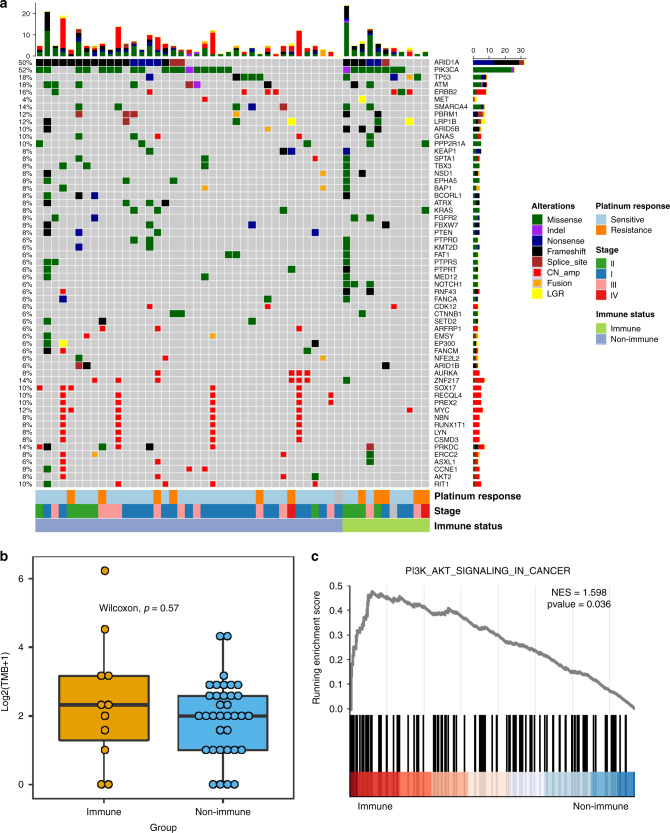
The significance of genomic mutations in the two immune subtypes of OCCC. **a** Selected significantly mutated genes (SMGs) in OCCC samples. The upper plot shows the frequency of mutation for each tumour sample. The central plot shows the types of mutations in each tumour sample (when the sample showed more than one mutation in the same gene, only the most deleterious type is shown). The lower part of the figure shows the response to platinum therapy, tumour stage and immune status of each sample. **b** Tumour mutation burden (TMB) distributions between the two groups. *P* value was based on Wilcox test. The middle line in the box is the median, the bottom and top of the box are the first and third quartiles, and the whiskers extend to 1.5× interquartile range of the lower and the upper quartiles, respectively. **c** Enrichment of PI3K-AKT pathway. OCCC = ovarian clear cell carcinoma.

A total of 563 somatic mutations were detected, spanning 236 genes in 50 patients. Figure 3a displays the distribution of gene alterations in the cohort. In line with previous studies, the most common mutant genes were ARID1A (50%) and PIK3CA (52%). Mutations were also frequently observed in other genes including TP53 (18%), ATM (18%), SMARCA4 (14%) and PRKDC (14%).

We proceed to investigate the tumour mutation burden (TMB) in OCCC. The entire cohort has low mutation burden (average 4.36), which is not surprising. We did notice one case with quite high TMB (73.78) in the immune subgroup. However, no significant difference was observed between the two subgroups (Fig. 3b). We further compared the genomic alterations based on immune subtypes. Interestingly, we observed that patients with the immune subtype had a higher mutation rate of PIK3CA (72.7% vs. 46.2%, Fig. 3a), although significance was not achieved (*P* = 0.11, Pearson Chi-square). Besides, the PI3K-AKT pathway did enrich in the immune subtype (Fig. 3c). No other correlation was observed between genomic alterations and immune classification.

### Construction and validation of a prognostic immune signature for OCCC patients

**Fig. 4:**
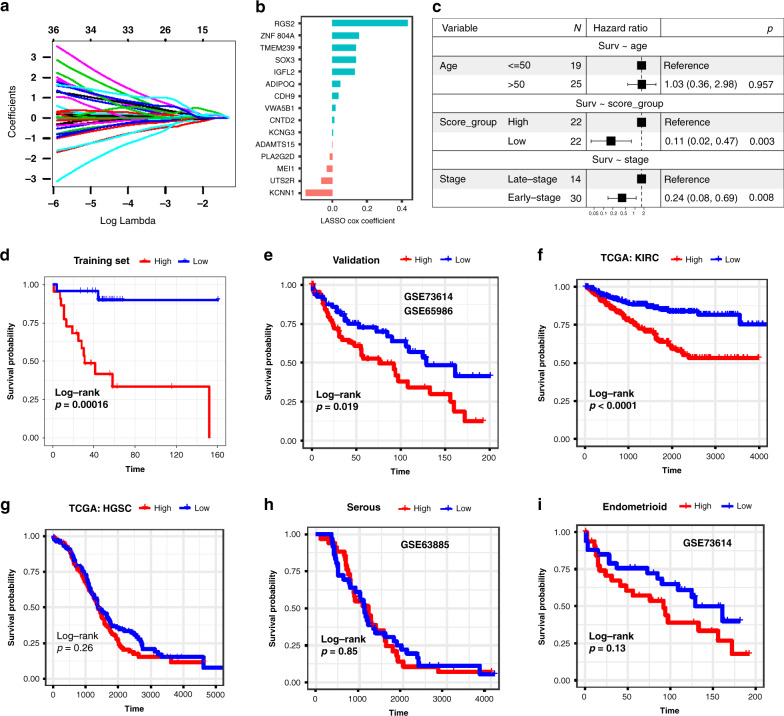
Establishment and validation of a prognostic immune signature for ovarian clear cell carcinoma (OCCC) patients. **a** LASSO Cox regression algorithm was used to identify the most robust prognostic genes. **b** An ensemble of 15 genes remained with individual coefficients. **c** Hazard ratio (HR) for overall survival analysis in the OCCC datasets. The solid squares denote the odds ratios (ORs) or risk difference (RD), and the horizontal lines represent the 95% confidence intervals (CIs). **d**, **e** Kaplan–Meier analysis demonstrated that patients with higher immune score exhibited worse overall survival in the training cohort (samples from our institution) (**d**) and validation cohort (**e**). **f** Survival analyses of lasso-cox models in TCGA-KIRC (kidney clear cell carcinoma). Kaplan–Meier analysis also demonstrated that patients with higher immune score exhibited worse overall survival. **g**–**i** Higher immune score exhibited worse overall survival was not for all kind of ovarian cancers, such as TCGA-OV which is composed of ovarian high-grade serous carcinoma (**g**), ovarian serous carcinoma (**h**) and ovarian endometrioid carcinoma (**i**).

In the last step, we tried to construct a prognostic immune signature for OCCC patients based on our cohort (Fig. 4). Firstly, differential expression analysis was performed between immune and non-immune group. By Lasso Cox regression analysis, a total of 15 genes remained with individual coefficients and led to immune score (Fig. 4b). The immune score and FIGO stage proved to be independent prognostic factors for overall survival (Fig. 4c). What’s more, the immune score was even more robust than stage, with hazard ratio of 0.11 and 0.24, respectively. Patients with higher immune scores showed worse overall survival in both the training cohort (data from our institution) (Fig. 4d) and the validation cohort (GSE73614, GSE65986) (Fig. 4e). Interestingly, the immune classification is also an independent prognostic factor in renal clear cell carcinoma based on the TCGA data (Fig. 4f). However, the classification could not be applied to other histologic subtype of ovarian cancer including serous and endometrioid histology (Fig. 4g, h, i). To sum up, a prognostic immune signature was established and validated for OCCC patients and it might be peculiar to clear cell histology and merits further study.

## Discussions

The current study is, to the best of our knowledge, one of the few publications that integrates genomic and transcriptomic analysis in ovarian clear cell carcinoma patients with a relatively large sample size. Transcriptomic analysis of immune pathway reveals an immune subtype, representing 22% of OCCC, with enrichment of PD-1 and PI3K signalling. Surprisingly, the immune subtype is associated with significantly poor survival. Integration with genomic analysis shows that the immune subtype patients had a higher mutation rate of PIK3CA albeit with no significance. When we compared the clinicopathological features of the two groups, we noted that there was a trend to younger age and platinum resistance in immune group. However, significance was not achieved. Cautions should be taken in interpretation given the relatively small sample size. For now, we couldn’t deny the possible relationship, which merits further study. Lastly, a 15-gene immune score signature was constructed as prognostic model for OCCC patients based on our own data and further validated in public repository. It is worth mentioning that the signature could be applied to renal clear cell carcinoma but not other histologic subtype of ovarian cancer.

Our first question was a simple but clinically important one, namely could we find any biological prognostic biomarkers inherent to the tumour? In a previous study, two transcriptomic subtypes (epithelial-like and mesenchymal-like) were identified by unsupervised gene expression analysis in OCCC [16]. The mesenchymal-like group was associated with advanced-stage, higher-enrichment of immune-related pathway and poor survival, while the epithelial-like was related to early-stage tumour, a higher frequency of SWI/SNF complex mutations and favourable outcome [16]. However, the study was limited by completeness, accuracy and quality of the samples collected given that most data were curated and re-analysed from public database [16]. In contrast, our transcriptomic classification (immune/non-immune), generated from our own data, was not correlated with tumour stage. In other words, we might speculate that the immune/non-immune subtype might be the inherent feature of tumour and independent of stage. In both studies, we did notice that the subtype with enrichment of immune-related pathways had worse survival. In addition, our immune signature was reproducible in external ovarian and renal clear cell carcinoma cohorts, while not in other histologic subtype of ovarian carcinoma. Using TCGA dataset, Iglesia and colleagues assessed the immune cell infiltration and overall survival across 11 tumour types, including ovarian cancer [37]. They concluded that heterogeneous immune infiltrates were present in different cancers and typically portend a good survival [37]. However, the statistic significance was not achieved in their study for ovarian cancer comprising of different histologic subtypes [37]. In a recent publication, the immune cell infiltrations (CD3+, FOXP3+, CD8+ T cells, CD20+ B cells) and expressions of PD-1, PD-L1 and IDO1 were evaluated in a cohort of 162 OCCC tumours on a tissue microarray by multiplex immunohistochemistry [38]. They found that increased infiltrations of CD8+ T and macrophage were related to poor survival, while high expressions of PD-L1 and IDO-1 were associated with good survival [38]. Actually, our work extends but not contradicts the study. We clarified the immune cell components, immune-related genes and pathways based on transcriptomic analysis. However, the specific underlying mechanism remains to be elucidated why the immune subtype portends grave survival in OCCC. We tried to get some hint from the coexistence of PIK3CA mutation and enrichment of PD-1 signalling in OCCC. In breast cancer, the PI3K pathway alterations might also be related to the high immune status [39]. In the exploratory analyses of a clinical trial of breast cancer, high CD8 infiltration was related to unfavourable survival and PI3K pathway alterations was correlated to the tumour microenvironment [40]. Activation of PIK3CA leads to formation of breast tumours with immune cell infiltration, as well as gene expression linked to Treg cell signalling and activation of targetable immune checkpoint pathways [41].

The second question of great concern was that could gene expression molecular subtypes provide any therapeutic hint for future study? There are no specific targeted treatments for OCCC, although many candidate targets have been identified [11]. Based on our study, we identified a high-risk immune subset, representing 22% of OCCC, with unfavourable survival. The immune subtype was characterised by high expression of PD-1 signalling, angiogenesis and PI3K/AKT/mTOR pathway. Interestingly, previous studies suggested that targeting PI3K/AKT/mTOR signalling could inhibit tumour progression by augmenting tumour immunosurveillance, preventing activation of immunosuppressive signalling and activating anti-tumour immunity [42, 43]. In addition, PI3K/AKT/mTOR signalling plays an important role in regulating angiogenesis and epithelial-mesenchymal transition [44, 45]. Therefore, further studies are warranted to elucidate the role of PI3K/AKT/mTOR signalling networks on immune regulation and tumour progression in OCCC. Recently, three phase II clinical trials focusing on persistent and recurrent OCCC patients showed that VEGF-R inhibitors (sunitinib [20], cabozantinib [46] and ENMD-2076 [47]) had minimal activity as a single agent. Therefore, we generated two hypotheses for future studies: (i) single-agent VEGF-R inhibitors might be more effective in the immune subset as single agent considering the transcriptomic analysis; (ii) the combination treatment might be more promising, including but no restricted in PI3K pathway targeted therapy, anti-angiogenesis agent, and checkpoint inhibitors. On the other hand, immune therapy seemed promising in OCCC despite that only small cases were included in previous trials. Two major questions remain to be answered. Firstly, is immunotherapy really effective in recurrent OCCC patients? If yes, as a single agent or in combination? Secondly, is there any predictive biomarkers for immunotherapy in OCCC patients? We tried to answer the latter question in detail. The well-established biomarkers for immunotherapy are PD-L1 expression, microsatellite instability (MSI) and mismatch repair deficiency (dMMR) and TMB [48]. From our study, we observed that immune subtype had higher mutation rate of PIK3CA. We wondered whether patients with PIK3CA mutation, which is one of the most common mutant genes in OCCC, might be predictive biomarkers for PD-1 inhibitor combination therapy. In the recent secondary analysis of the CLAP trial, the PIK3CA mutation was found to be a novel predictive biomarker in cervical cancer patients treated with combination therapy of PD-1 and VEGF-R inhibitor [49]. Similarly, the RTK/Ras/PI3K/AKT pathway alterations might be potential biomarkers for immunotherapy in diffuse gliomas [50].To better answer the above questions, we genuinely hope to initiate prospective clinical trials to further explore the role of immune cluster as a predictive marker for immunotherapy and/or other biologic agents.

The present study has several limitations. Firstly, given disease rarity, we only include 50 cases. The sample size might be relatively large as compared to other single-institutional study focusing on OCCC. However, the sample size largely limits the statistical power for subgroup analyses and cautions should be taken when interpreting the results. Secondly, the limited scope of multi-gene panels might affect the ability to evaluate overall mutation burden and patterns, signatures that might be relevant for neo-antigen formation. Thirdly, some clinicopathological information was missing due to the retrospective study design. Fourthly, the clinical relevance and predictive role of PIK3CA mutation in immunotherapy lack functional validation in pre-clinical or clinical models. Lastly, we only included platinum-response in the study and no statistical association between immune subtype and platinum sensitivity was found given the current small sample size. In the near future, we hope to initiate a prospective clinical trial to further explore the role of immune cluster as a predictive marker for immunotherapy and/or other biologic agents.

## Conclusions

By integrative genomic and transcriptomic analysis, we delineated two different OCCC molecular subtypes with different functions and prognosis. The immune subset was enriched in PD-1 and PI3K-AKT-mTOR signalling. A robust prognostic immune signature of OCCC patients was constructed based on our data and reproducible in public repositories. The predictive role of immune/non-immune classification to target therapy merits and awaits further prospective studies.

## Supplementary information

supplementary legends

Supplementary figure S1: Kaplan–Meier curves for overall survival (OS) in external validation cohorts.

Supplementary figure S2: Kaplan–Meier curves based on immune/non-immune subtype in other ovarian histologic cancers and renal clear cell carcinoma.

Supplementary Table S1: The specific details of 520-gene panel.

Supplementary Table S2: Univariate and multivariate Cox regression analysis of survival in ovarian clear cell carcinoma by different molecular subtypes.

## Data Availability

The dataset supporting the conclusions of this article is available upon request. Please contact Dr Huijuan Yang (huijuanyang@hotmail.com).
