# Supplementary material for: Integrative genomic and transcriptomic analysis reveals immune subtypes and prognostic markers in ovarian clear cell carcinoma
Source: Br J Cancer. 2022 Jan 18;126(8):1215–23. doi: 10.1038/s41416-022-01705-w (PMC9023449; doi:10.1038/s41416-022-01705-w)
Supplement: Supplementary file 1 — supplementary legends [file 41416_2022_1705_MOESM1_ESM.docx]

**Supplementary figure legends**

Supplementary figure S1: Kaplan–Meier curves for overall survival (OS) in external validation cohorts. (A) GSE73614 (Caucasian, n=37). (B) GSE65986 (Japanese, n=25). (C) The two cohorts were amalgamated given the small sample sizes and outcome events. Ovarian clear cell carcinoma patients with immune subtype had worse overall survival.

Supplementary figure S2: Kaplan–Meier curves based on immune/non-immune subtype in other ovarian histologic cancers and renal clear cell carcinoma. (A) TCGA: KIRC (kidney renal clear cell carcinoma. The classification could be reproduced in patients with KIRC. (B) Ovarian endometrioid cancer. (C) Ovarian high-grade serous carcinoma. The classification could not be applied to ovarian endometrioid or high-grade serous carcinoma.

**Supplementary table legends**

Supplementary Table S1: The specific details of 520-gene panel.

Supplementary Table S2: Univariate and multivariate Cox regression analysis of survival in ovarian clear cell carcinoma by different molecular subtypes.
