# Supplementary figures and images for: Integrative genomic and transcriptomic analysis reveals immune subtypes and prognostic markers in ovarian clear cell carcinoma
Source: Br J Cancer. 2022 Jan 18;126(8):1215–23. doi: 10.1038/s41416-022-01705-w (PMC9023449; doi:10.1038/s41416-022-01705-w)

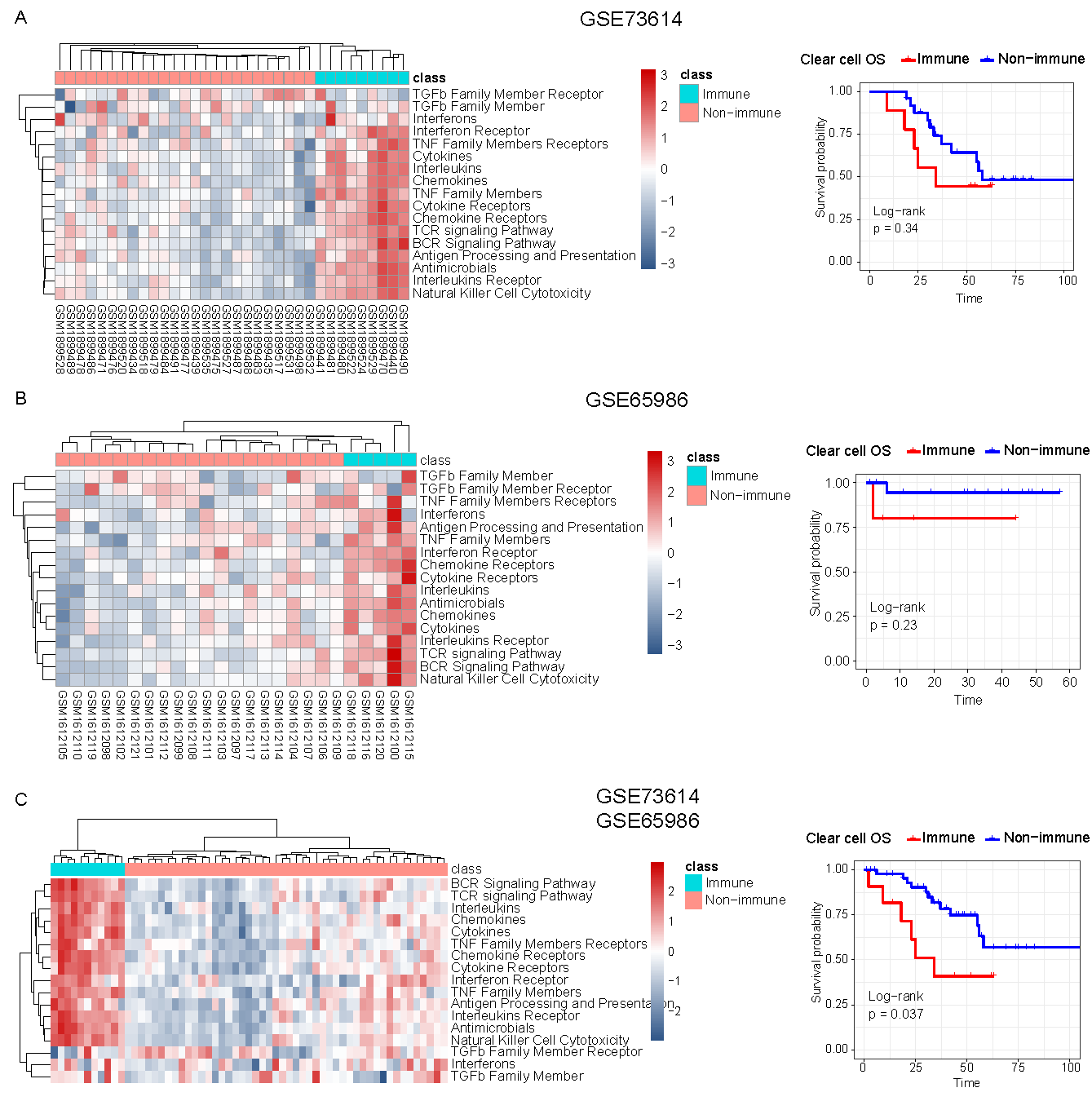

Supplement: Supplementary file 2 — Supplementary figure S1: Kaplan–Meier curves for overall survival (OS) in external validation cohorts. [file 41416_2022_1705_MOESM2_ESM.tif]

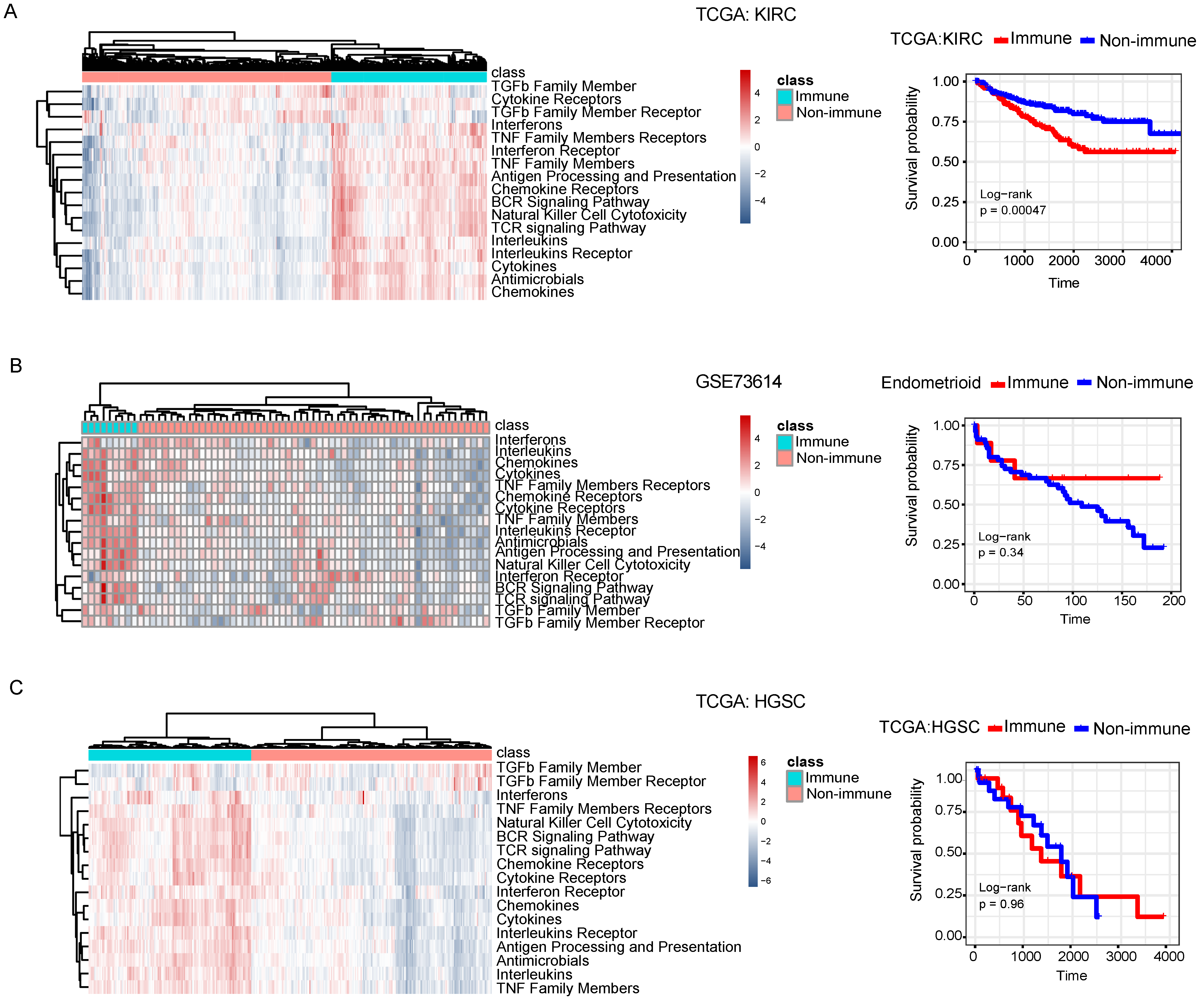

Supplement: Supplementary file 3 — Supplementary figure S2: Kaplan–Meier curves based on immune/non-immune subtype in other ovarian histologic cancers and renal clear cell carcinoma. [file 41416_2022_1705_MOESM3_ESM.tif]
